# Diagnostic values of serum cathepsin B and D in patients with nasopharyngeal carcinoma

**DOI:** 10.1186/s12885-016-2283-4

**Published:** 2016-03-19

**Authors:** Gongjun Tan, Qianxu Liu, Xiaowei Tang, Ting Kang, Yuejin Li, Jinping Lu, Xiaoming Zhao, Faqing Tang

**Affiliations:** Department of Clinical Laboratory and Medical Research Center, Xiangya Hospital, Central South University, 87 Xiangya Road, Changsha, 410008 Hunan China; Department of Clinical Laboratory and Medical Research Center, Zhuhai People’s Hospital, Zhuhai Hospital of Jinan University, 79 Kangning Road, Zhuhai, 519000 Guangdong China; Metallurgical Science and Engineering, Central South University, 21 Lushan South Road, Changsha, 410083 China; State Key Laboratory of Oncology in South China, Sun Yat-sen University Cancer Center, Guangzhou, China

**Keywords:** Biomarker, Diagnostic value, Nasopharyngeal carcinoma, Cathepsin B, Cathepsin D

## Abstract

**Background:**

The diagnostic and prognostic significance of increased cathepsin B (CTSB) and cathepsin D (CTSD) concentration in the serum of cancer patients were evaluated for some tumor types. High expression of CTSD and CTSB was detected in biopsy tissues from nasopharyngeal carcinoma (NPC). However, whether CTSD and CTSB serve as diagnostic and prognostic markers of NPC remains unclear.

**Methods:**

Serum samples were collected from 40 healthy volunteers and 80 NPC patients enrolled in the study. CTSB and CTSD in the serum samples were detected using enzyme-linked immunosorbent assay (ELISA). Concomitantly, the relationship between CTSB and CTSD concentrations and clinicopathological prognosis was assessed. The sensitivity and specificity of the two components in the diagnosis of NPC were evaluated in 80 NPC patients.

**Results:**

ELISA analysis showed that in the sera obtained from NPC patients, the CTSB concentration was 12.5 ± 3.5 mg/L (median, 12.4 mg/L), and the CTSD concentration was 15.7 ± 8.7 mg/L (median, 14.7 mg/L). CTSB and CTSD levels were significantly higher in the NPC patient population compared to the healthy control population (*p* = 0.001; *p* = 0.001, respectively). The presence of CTSB and CTSD in the serum of the patients with NPC correlated with the tumor node metastasis (TNM) scores (*p* = 0.001). Other parameters were not identified to be of significance. Receiver operating characteristic (ROC) analysis showed that a cut off CTSB concentration of 12.4 mg/L had 61.9 % sensitivity and 63.2 % specificity in the prediction of progression-free survival (Area under the curve (AUC) = 0.525; 95 % CI, 39.7–65.2; *p* = 0.704); whereas a cut off CTSD concentration of 14.7 mg/L had 66.7 % sensitivity, and 58.5 % specificity (AUC = 0.552; 95 % CI, 42.3–68.1; *p* = 0.42).

**Conclusions:**

Serum CTSB and CTSD concentrations were found to have a diagnostic value in NPC. However, the CTSB and CTSD serum levels had no prognostic role for the outcome in NPC patients.

## Background

Nasopharyngeal carcinoma (NPC) is one of the most common malignancies in Southern China, Southeastern Asia, and Northern Africa. The lowest prevalence of NPC is observed in white populations from Europe and the United States [1]. Most patients are initially diagnosed at a late stage, resulting in high mortality rates. Radiotherapy is currently the primary treatment modality for patients with NPC. Although the tumor node metastasis (TNM) staging system is currently the most powerful prognostic factor of NPC, patients with the same TNM stage undergoing similar treatment regimens show variable clinical outcomes [2]. However, locoregional recurrence and distant metastasis following radiotherapy still have deleterious effects on the survival rates of patients with NPC [3]. Individualized therapy is needed to improve long-term survival and quality of life [2]. Determining clinical and biological characteristics of NPC, and identifying markers that can predict prognosis might make individualization of treatment possible [4].

Cathepsins are a class of globular proteases that were initially described as intracellular peptide hydrolases, although several cathepsins also have extracellular functions [5]. Cathepsins become proteolytically activated when attached to other cell surface proteins [6]. This extracellular activity enables cancer cell invasion of surrounding tissues, blood, and lymph vessels, and metastasis to distant sites. Numerous studies have shown a correlation between cathepsin proteolytic activity and neoplastic transformation, tumor invasion, and metastasis through the destruction of extracellular matrix components and basement membranes [7]. Elevated cathepsin B (CTSB) and cathepsin D (CTSD) levels were detected in primary and metastatic tumor tissues of various cancer types [8]. Elevation of CTSB and CTSD levels in biological fluid has been observed in patients with inflammatory diseases and many cancers. CTSB and CTSD are members of the cathepsin family. CTSB is a lysosomal cysteine protease of the papain family of enzymes that function as endopeptidases and exopeptidases [9]. CTSD is from the family of aspartic proteases that function in intracellular catabolism at lysosomal compartments; other physiological effects include hormone and antigen processing [10, 11]. While they can both degrade laminin, fibronectin, collagen, and other extracellular matrix components, and promote the formation of tumor blood vessels [12], there is no similarity in their amino acid sequences. Our previous studies showed that CTSB and CTSD are highly expressed in NPC metastatic tissues, and they increased cell motility and invasion, and promoted NPC tumor metastasis [13]. Therefore, we speculate that CTSB and CTSD may be biomarkers of NPC metastasis, and could be of prognostic significance for NPC metastasis. In this study, we detected the concentrations of CTSB and CTSD in the sera of NPC patients and healthy controls using ELISA, analyzed the correlation of CTSB and CTSD concentrations with NPC progression, and evaluated the diagnostic significance of serum CTSB and CTSD concentrations for NPC patients. Our results showed that serum CTSB and CTSD concentrations are of diagnostic significance for NPC patients; however, they have no prognostic value in NPC patient outcomes.

## Methods

The study protocol was approved by Ethical Committee at Zhuhai Hospital of Jinan University, and the written informed consent was obtained from each patients and control subjects. The participants declared that their sample and data are only used for this research in the signed informed consent, and the data could not be used in other research. The procedures of the study followed were in accordance with the ethical standards of the committee on human experimentation of the institution.

### Patient samples

Eighty consecutive NPC patients (52 men and 28 women) with a median age of 45 years (range, 16–77), admitted to Sun Yat-sen University Cancer Center between February 2011 and December 2012, were enrolled in this study. The patients’ characteristics with respect to age, sex, and ethnic origin were recorded. All of the enrolled patients were uniformly given a routine diagnostic work up constituted of a detailed clinic head and neck examination, nasopharyngoscopy, and the histological and cytological examination of tumor tissue. Tumor histology and stages were classified according to the World Health Organization (WHO) classification and the TNM staging system of Union for International Cancer Control (UICC), respectively. The concentrations of bilirubin, hepatic enzymes, and markers of renal function were also analyzed. The control group consisted of 40 healthy relatives of the NPC patients (20 men and 20 women), with a median age of 45 years (range, 20–78 years), and no known diseases.

### Sample collection

Blood samples were obtained from fasting subjects under standard conditions. During the study period, blood samples were collected by venipuncture from the healthy control and patient population everyday between 9: 00 AM and 10: 00 AM. Samples were clotted at 4–8 °C, and then centrifuged at 3000 rpm for 10 min. The collected serum was distributed in aliquots of 500 μL each, and stored at − 80 °C until required.

### Reagents and kits

Chemical reagents used in the preparation of phosphate-buffered saline (PBS), including NaCl, KCl, Na_2_HPO_4_, and KH_2_PO_4_, were purchased from Sigma-Aldrich (St.Louis, MO). These salts were dissolved in 800 mL of distilled water, and the pH adjusted to7.4 with HCl. Distilled water was added up to a final volume of 1 L. The resultant 1 × PBS should have a final concentration of 10 mM PO_4_^3−^, 137 mM NaCl, and 2.7 mM KCl. CTSB and CTSD enzyme-linked immunosorbent assay (ELISA) kits were purchased from Wuhan Eiaab Science Co. Ltd (Hubei, China).

### Measurement of CTSB and CTSD

Using commercial CTSB and CTSD ELISA kits, CTSB and CTSD detection was performed according to the manufacturer’s instructions. All samples were detected simultaneously. Serum concentrations of CTSB and CTSD were determined using standard curves and expressed as units per liter (mg/L). The linear ranges for CTSB and CTSD were 0–50 mg/L and 0–100 mg/L, respectively.

### Statistical analysis

The group differences were assessed using nonparametric tests. Median concentrations of CTSB and CTSD in the patient and healthy control groups were compared using Mann–Whitney *U* test. Using the median values as cut off values for both markers, the receiver operating characteristic (ROC) analysis was performed. The significance of serum CTSB and CTSD concentrations in the prediction of progression-free survival (PFS) was evaluated by univariate analysis. Survival curves were constructed using the Kaplan–Meier method, and compared with log-rank tests. Factors predictive of relapse were analyzed by both univariate and multivariate analyses using a Cox proportional hazards model. Multivariate *p* values were used to characterize the independence of these factors. The relationship between survival time and each independent factor was quantified by calculating the 95 % confidence interval (CI). All *p* values were two-sided, and *p* values < 0.05 were considered statistically significant. All statistical analyses were performed using SPSS 17.0.

## Results

The median ages for the control and patient groups were 40 years (range, 23–71) and 52.5 years (range, 18–86), respectively. Among 80 patients, 22 (27.5 %) had stage II, 25 (31.25 %) had stage III, and 33 (41.25 %) had stage IV diseases. Seventy patients (87.5 %) were diagnosed with undifferentiated carcinoma. Sixty-eight patients (85.0 %) completed radical radiotherapy. Demographic, disease, treatment, and relapse characteristics of the patients are summarized in Table 1.

**Table 1:** Demographic, disease, treatment and relapse characteristics of the patients with nasopharyngeal carcinoma

Variables	Number (%)
Median Age (min–max)	45 (16–77)
Sex	
Men	52 (65)
Women	28 (35)
Stage	
II	22 (27.5)
III	25 (31.25)
IV	33 (41.25)
Pathology	
Differentiated	10 (12.5)
Undifferentiated	70 (87.5)
Treatment	
Yes	68 (85)
No	12 (15)
Relapse	
None	56 (70)
Distant	12 (15)
Locoregional	6 (7.5)
Distant and Locoregional	6 (7.5)

CTSB and CTSD were identified to be associated with various cancer types [8], and we previously showed that CTSB and CTSD play important roles in NPC development and metastasis [13]. To evaluate the value of CTSB and CTSD in the clinical diagnosis of NPC, at first, CTSB and CTSD were detected in the sera of NPC patients and the healthy population. The results showed that median serum CTSB was 12.5 ± 3.5 mg/L in the NPC group and 2.5 ± 1.4 mg/L in the healthy control; while CTSD concentration was 15.7 ± 8.7 mg/L in the NPC group and 2.7 ± 0.8 mg/L in the healthy control population. CTSB and CTSD were significantly higher in the NPC group than in the healthy control group (*p* < 0.001) (Table 2).

**Table 2:** Serum CTSB and CTSD concentrations of the patients with nasopharyngeal carcinoma and the healthy population

	Patients (*n* = 80)		Controls (*n* =40)		*p* value
	Mean (±SD)	Median (min–max)	Mean (±SD)	Median (min–max)	
CTSB (mg/L)	12.5 ± 3.5	12.4 (4.4–25.6)	2.5 ± 1.4	2.1 (0.6–5.0)	0.001
CTSD (mg/L)	15.7 ± 8.7	14.7 (2–42.3)	2.7 ± 0.8	2.8 (1.3–3.7)	0.001

*Notes*: *CTSB* cathepsin, B *CTSD* cathepsin D, *SD* standard deviation

The associations between CTSB and CTSD concentrations and patients’ clinical characteristics were further analyzed. The data showed a significant relationship between CTSB and CTSD concentrations and TNM grade (*p* = 0.0001 and *p* = 0.002, respectively). We did not observe any significant relationships between CTSB and CTSD concentrations, and patient age, sex, viral capsid antigen (VCA)-IgA, alcohol intake, and smoking status (Table 3).

**Table 3:** Relationships between serum CTSB and CTSD concentrations and clinicopathological characteristics of patients with nasopharyngeal carcinoma

Variables	Number	CTSB (mg/L)	*P* value	CTSD (mg/L)	*p* value
		Median (min–max)		Median (min–max)	
Sex					
Men	52	12.4 (4.4–25.6)	0.823	16.6 (2–42.3)	0.153
Women	28	12.0 (8.0–19.0)		2.7 (4.6–29.1)	
Age, years					
≤ 45	42	11.9 (4.4–25.6)	0.855	15.3 (3.8–42.3)	0.086
> 45	38	12.5 (7.8–19.0)		14.4 (2–28.7)	
Smoking					
Yes	55	12.4 (6.5–25.6)	0.583	14.7 (3.8–42.3)	0.519
No	25	12.5 (4.4–19.0)		15.5 (2–29.6)	
Alcohol intake					
Yes	57	12.5 (5.0–25.6)	0.675	14.4 (4.2–42.3)	0.985
No	23	12.4 (4.4–17.5)		15.3 (2–33.4)	
TNM grade					
I + II	22	9.2 (4.4–17.3)	0.0001	8.7 (2.0–24.7)	0.002
III + IV	58	13.1 (8.9–25.6)		15.7 (2.8–42.60)	
EBV/VCA-IgA					
≤ 1:160	44	11.9 (6.5–25.6)	0.693	14.9 (4.2–33.4)	0.441
≥ 1:320	36	12.6 (4.4–19.0)		12.6 (2.0–42.6)	

*Notes*: *CTSB* cathepsin B, *CTSD* cathepsin D, *TNM* tumor node metastasis, *EBV* Epstein-Barr virus, *VCA-IgA* viral capsid antigen IgA

**Fig. 1:**
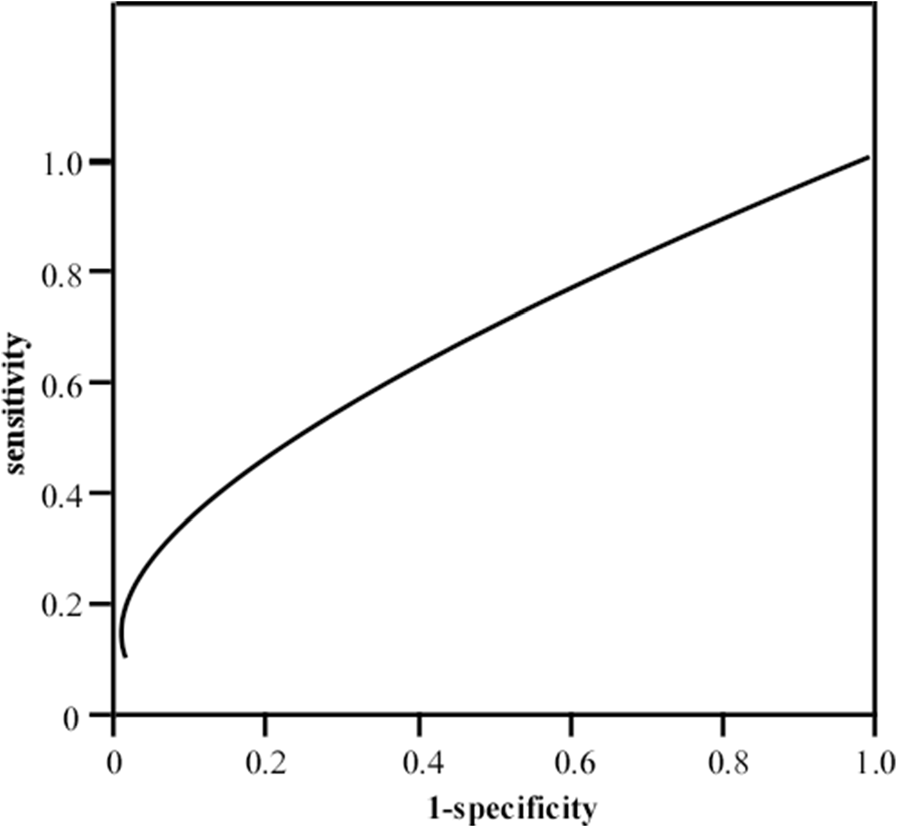
Receiver operating characteristic (ROC) analysis of cathepsin B (CTSB). The area under the curve (AUC) of CTSB is 0.525 [95 % confidence interval (CI) 39.7 – 65.2]. Cutoff value of 12.4 mg/L had a sensitivity of 61.9 % and a specificity of 63.2 %

**Fig. 2:**
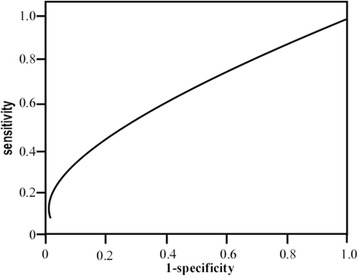
Receiver operating characteristic (ROC) analysis of cathepsin D (CTSD). The area under the curve (AUC) of CTSD is 0.552 [95 % confidence interval (CI) 42.3–68.1]. Cutoff value of 14.7 mg/L had a sensitivity of 66.7 % and a specificity of 58.5 %

The significance of CTSB and CTSD concentrations in the prediction of NPC progression-free survival (PFS) was assessed. The median follow-up period for NPC patients was 24 months. During follow-up, 24 patients developed disease recurrence, including 12 with distant metastasis, 6 with local regional relapse, and 6 with both (Table 1). The 1-year PFS rate was 78.5 %, whereas the median PFS was 25.6 months (min–max: 1.8–46.5). For each of the two parameters, overall survival was compared in patients with levels below, and equal to or above the median. We used ROC analysis to evaluate the prognostic significance of CTSB and CTSD concentrations for PFS, and identified that a CTSB cutoff value of 12.4 mg/L had a sensitivity of 61.9 % and a specificity of 63.2 % (AUC = 0.525;95 % CI, 39.7–65.2; *p* = 0.704) (Fig.1), whereas a CTSD cutoff value of 14.7 mg/L had a sensitivity of 66.7 % and a specificity of 58.5 % (AUC = 0.552; 95 % CI, 42.3–68.1; *p* = 0.42) (Fig.2). However, they were not significant prognostic factors for PFS. Univariate analysis also showed that clinical stage (*p* = 0.5), patient age (*p* = 0.7), sex (*p* = 0.9), VCA-IgA (*p* = 0.7), and smoking status (*p* = 0.9) were not associated with PFS. Similarly, multivariate analysis showed that serum CTSB or CTSD concentrations were not of prognostic significance for PFS (Table 4).

**Table 4:** Univariate and multivariate analyses of CTSB and CTSD for prognosis of patients with nasopharyngeal carcinoma

Variables	Number	Median PFS	Univariate analysis	Multivariate analysis	
		(months)	*p* value	*p* value	RR
CTSB					
> 12.4 mg/l	42	37.0	0.699	0.878	0.938
≤ 12.4 mg/l	38	37.8			
CTSD					
> 14.7 mg/l	39	35.9	0.216	0.341	1.524
≤ 14.7 mg/l	41	38.6			
Sex					
Men	54	37.2	0.902	0.566	1.286
Women	26	35.8			
Age					
≤ 45 years	40	37.3	0.699	0.779	0.892
> 45 years	40	37.5			
TNM stage					
I + II	20	39.1	0.454	0.556	1.376
III + IV	60	36.4			
VCA-IgA					
≤ 1:160	45	37.5	0.735	0.769	1.265
≥ 1:320	35	36.8			
Smoking					
Yes	55	36.6	0.913	0.991	0.995
No	25	37.9			

*Notes*: *CTSB* cathepsin B, *CTSD* cathepsin D, *PFS* progression-free survival, *RR* relative risk, *TNM* tumor node metastasis, *EBV* Epstein-Barr virus, *VCA-IgA* viral capsid antigen-IgA

## Discussion

A number of clinical studies have been performed to evaluate the diagnostic and prognostic significance of elevated CTSB and CTSD concentrations in tumor cytosols [14]. It was shown that patients with higher concentration or increased proteolytic activity of CTSB in primary lung tumors exhibited significantly higher risk of recurrence or death compared to patients with a low concentration of the enzyme [15]. CTSD and CTSB activities in the sera of patients with urothelial bladder cancer were identified to be directly proportional to disease severity, and were significantly higher compared to those of the control group [16]. The elevation of CTSB and CTSD concentrations was shown in the sera of patients with some tumor types, including colorectal cancer, and bladder cancer [16, 17]. We previously showed that CTSD and CTSB are highly expressed in NPC tissue biopsies [13]. However, whether CTSD and CTSB serve as diagnostic markers of NPC has not been investigated. In this study, we detected the CTSD and CTSB concentrations in the sera of NPC patients and healthy controls, and analyzed the relationship between CTSB and CTSD concentrations and the occurrence of NPC. Our data showed that the CTSB and CTSD concentrations were higher in the sera of the NPC patients than in those of the healthy controls. Based on these results, we speculated that CTSB and CTSD concentrations might increase with the progression of NPC. In order to validate, we comparatively assessed the relationship of CTSB and CTSD with the NPC TNM grade for significance. The results showed that the concentrations of CTSB and CTSD increase with increasing TNM grade. We hypothesize that CTSB and CTSD might serve as candidate biomarkers of NPC.

Retrospective immunohistochemical analysis showed that lung cancer patients with upregulated CTSB tend to have higher rates of hematogenous and intrapulmonary metastases [18]. CTSB activity is significantly elevated in a variant of B16 melanoma with tumor cells of high metastatic potential [19]. Intense expression of CTSD in high-grade carcinomas might be an indicator of invasive potential and aggressive behavior [20]. Among the patients with positive lymph nodes, those with tumor cells immunopositive for CTSD had a higher risk of relapse [21]. Determination of CTSD status in breast cancer patients might help identify those with different risk levels of relapse [22]. Our previous work suggests that CTSB and CTSD are associated with NPC metastasis [13]. Next, we analyzed whether CTSB and CTSD concentrations increase in the sera of metastatic NPC patients. The data showed that CTSB and CTSD levels were significantly higher in the sera of patients with metastatic NPC than in the sera of patients with no metastasis. Therefore, the detection of CTSB and CTSD concentrations might indicate the prognosis for NPC metastasis.

Epstein-Barr virus (EBV) infection is an important etiological factor for NPC [23]. EBV serological tests, including IgA for early antigen (EA) and VCA, have been used for NPC detection for a long time [24, 25]. We analyzed whether CTSB and CTSD concentrations are related to EBV infection in NPC patients. We did not observe any significant relationship between CTSB and CTSD concentrations, and EBV infection. We did not yet identify any relationship between CTSB and CTSD concentrations, and patient age, sex, alcohol intake, and smoking status.

CTSB and CTSD expression was detected in NPC and normal nasopharyngeal epithelial tissues, and the results showed that CSTB and CTSD expression levels were significantly higher in the NPC tissues compared to the normal nasopharyngeal epithelial tissues. Further analysis showed that the upregulation of CTSB or CTSD was significantly correlated with lymph node metastasis, advanced clinical stage, recurrence, distant metastasis, and poor prognosis [13, 26]. Altogether, it is suggested that the serum concentrations of CTSB and CTSD might be potential biomarkers of NPC. Unfortunately, data enabling a definitive discrimination is currently not available.

## Conclusions

In summary, serum CTSB and CTSD concentrations were significantly higher in patients with advanced NPC, indicating that they could be diagnostic biomarkers of NPC. CTSB and CTSD serum levels are of prognostic significance in NPC progression. Future prospective studies with larger patient numbers and longer follow-up periods are required to further evaluate the prognostic significance of these potential biomarkers.

## Abbreviations

AUC: area under the curve
CI: confidence intervals
CTSB: cathepsin B
CTSD: cathepsin D
EA-IgA: IgA for early antigen
EBV: Epstein-Barr virus
ELISA: enzyme-linked immunosorbent assay
NPC: nasopharyngeal carcinoma
PBS: phosphate-buffered saline
PFS: progression-free survival
ROC: receiver operating characteristic
TNM: tumor node metastasis
VCA-IgA: viral capsid antigen IgA.
